# Correlation between peripheral blood lymphocyte subpopulations and primary systemic lupus erythematosus

**DOI:** 10.1515/biol-2022-0093

**Published:** 2022-08-10

**Authors:** Yan Feng, Zhijun Li, Changhao Xie, Fanglin Lu

**Affiliations:** Rheumatology Department, First Affiliated Hospital of Anhui University of Science and Technology, Huai Nan, China; Rheumatology Department, First Affiliated Hospital of Bengbu Medical College, Bangbu, China

**Keywords:** systemic lupus erythematosus, lymphocyte, disease activity, flow cytometry, complement C3

## Abstract

This study explored the correlation between peripheral blood CD3^＋^, CD3^＋^/CD4^＋^, CD3^+^/CD8^+^, CD4^＋^/CD8^＋^, CD3^−^/CD16^+^ CD56^+^, and CD3^−^CD19^+^ and disease activity of different subtypes of systemic lupus erythematosus (SLE). The percentages of CD3^+^, CD3^+^/CD4^+^, CD3^+^/CD8^+^, CD4^+^/CD8^+^, CD3^−^/CD16^+^ CD56^+^, and CD3^−^CD19^+^ in the peripheral blood of patients (*n* = 80) classified into lupus nephritis, blood involvement, and joint involvement and SLE in different active stages were detected by flow cytometry. Their correlations with baseline clinical experimental indicators of SLE patients’ SLE disease activity index score (SLEDAI) and complement C3 were analyzed. The results showed that CD3^+^, CD3^+^/CD4^+^, and CD3^+^/CD8^+^ at baseline level were negatively correlated with SLEDAI scores. These were positively correlated with C3. In conclusion, T-lymphocyte subpopulations are closely related to SLE activity and can be used as reference indicators to evaluate the SLE activity.

## 1 Introduction

Systemic lupus erythematosus (SLE) is an autoimmune disease characterized by excessive B cells and an imbalance of T and natural killer (NK) lymphocytes. In addition to T, B, and NK lymphocytes in SLE, there are disorders and mediators of cytokine communication that can be used as prognostic markers of the disease [1,2]. It involves multiple organs and systems, such as the skin, joints, blood, kidneys, and respiratory system, and its pathogenesis is not yet fully understood [3]. Dysregulated lymphocyte subpopulation ratios and abnormal immune cytokine expression are associated with the paroxysm of SLE and might be major factors in the pathogenesis of SLE [4,5]. This study examined the correlations between the expression percentage of T, NK, and B lymphocytes in the peripheral blood and the disease activity of 80 patients with different subtypes of SLE. In addition, the correlation between the differences in the expression of lymphocyte subpopulations in various clinical subtypes of SLE and the clinical laboratory indexes, such as SLE disease activity index (SLEDAI) score, complement C3, complement C4, IgG, IgA, IgM, erythrocyte sedimentation rate (ESR), and C-reactive protein (CRP), were analyzed. The present study aimed to identify the close correlation between lymphocyte subpopulations and SLE disease activity and provide a basis for the clinical diagnosis and treatment of the disease.

## 2 Materials and methods

### 2.1 General materials

The study collected clinical data from 80 in-patients with SLE from January 2019 to early June 2021 at our hospital. Inclusion criteria were as follows: (i) diagnosis in line with the 2019 EULAR/ACR SLE classification criteria [6]; (ii) SLEDAI score ≥5; and (iii) no steroid hormone or immunosuppressive therapy before inclusion. Exclusion criteria were as follows: (i) comorbidities such as other rheumatic immune diseases, tumors, and severe infections; (ii) SLEDAI score <5; and (iii) patients with SLE currently under standard treatment.


**Informed consent:** Informed consent has been obtained from all individuals included in this study.
**Ethical approval:** The research related to human use has been complied with all the relevant national regulations and institutional policies and in accordance with the tenets of the Declaration of Helsinki and has been approved by the First Affiliated Hospital of Anhui University of Science and Technology (2019074X).

### 2.2 SLEDAI score

Disease activity was assessed according to the SLEDAI 2K scoring system [7], with 0–4 being stable, 5–9 being mildly active, 10–14 being moderately active, and ≥15 being severely active.

### 2.3 Laboratory

On the second day after admission, 2 ml of fasting venous blood was taken in the morning and anticoagulated with EDTA. Two TruCount tubes were numbered A and B in sequence, and 50 µl of fully mixed anticoagulated whole blood was added into the tubes. Then, 10 µl of the CD3FITC/CD8PE/CD45PercP/CD4APC antibody was added into test tube A, and 10 µl of the CD3FITC/CD16 + 56PE/CD45 PercP/CD19APC antibody was added into test tube B. The tubes were vortexed, mixed evenly, and placed in a dark environment at room temperature for 15 min. Then, 450 µl of red blood cell lysate was added, fully mixed, and kept away from light at room temperature for 15 min. The cells were washed twice with PBS buffer and placed into a flow cytometry system (FC 500 MCL, Beckman Coulter, Brea, CA, USA) [8]. The flow cytometry laser was a cold sub laser with a wavelength of 488 nm. The light path was adjusted by the flow check. The voltage was adjusted. The lateral angular scattering (SSC) was the abscissa, and the forward angular scattering (FSC) was the ordinate. On the scatter diagram, the gating technique was used to analyze the percentage of total T cells (CD3^+^), CD4^+^ cells (CD3^+^/CD4^+^), CD8^+^ cells (CD3^+^/CD8^+^), NK cells, and B cells in the lymphocyte population. The ratio of the CD4^+^/CD8^+^ cells was calculated. The study collected complements C3 and C4, ESR, CRP, IgA, IgM, and IgG from 80 patients with primary SLE using immunoturbidimetric analysis on a BN-II-specific protein analyzer (Siemens, Erlangen, Germany).

### 2.4 Statistical analysis

The flow cytometry system software was CXP 2.0, and the statistical software for data analysis was SPSS 25.0. The normally distributed continuous data were expressed as mean ± standard deviation, and the *t*-test was used to compare groups. Correlations were analyzed using the Spearman test. The graphs were plotted using Spearman and GraphPad Prism software for correlation analysis. *P* < 0.05 indicated statistical significance.

## 3 Results

### 3.1 Basic clinical features of SLE patients

The cohort of 80 SLE patients consisted of four males and 76 females, with a male-to-female ratio of 1:19. The patients were 39.3 ± 13.9 (range: 15–75) years of age. The duration of the disease was 1–36 months. Among them, 55 cases had blood involvement (68.8%), 30 had lupus nephritis (LN) (37.5%), 36 had joint involvement (45%), 38 had skin involvement (47.5%), 6 had alopecia (7.5%), 16 had stomatitis (20%), 4 had pleural effusion (5%), 2 had pericardial effusion (2.5%), 19 had a fever (23.8%), and 1 had gastrointestinal vasculitis (1.3%).

### 3.2 Comparison between the LN and non-LN groups

The expression percentages of peripheral blood T cells (CD3^+^/CD4^+^ and CD4^+^/CD8^+^) and NK lymphocytes (CD3^−^/CD16^+^ CD56^+^) in the LN group were lower than in the non-LN group. On the other hand, the peripheral blood T cells (CD3^+^/CD8^+^) were higher than those in the non-LN group (*P* < 0.05). Moreover, the percentages of CD3^+^ and CD3^−^/CD19^+^ did not differ significantly in the two groups (*P* > 0.05; Table 1).

**Table 1:** Results of peripheral blood lymphocyte subpopulation testing in the LN and non-LN Groups of SLE (mean ± standard deviation)

Group	*N*	CD3^+^ (%)	CD3^+^/CD4^+^ (%)	CD3^+^/CD8^+^ (%)	CD4^+^/CD8^+^	CD3^−^/CD16^+^CD56^+^ (%)	CD3^−^/CD19^+^ (%)
LN	30	72.17 ± 7.13	25.27 ± 3.99	46.57 ± 6.3	0.55 ± 0.11	6.13 ± 2.05	6.17 ± 2.07
Non-LN	50	69.56 ± 6.36	28.64 ± 5.11	40.52 ± 3.97	0.71 ± 0.17	9.08 ± 1.96	6.4 ± 1.87
*t*		1.646	−3.286	4.727	−4.917	−6.337	−0.506
*P*		0.105	0.002	0.001	0.001	0.001	0.615

### 3.3 Comparison between the blood system involvement and no blood system involvement groups

Peripheral blood T cells (CD3^+^/CD4^+^ and CD4^+^/CD8^+^) in the blood system involvement group were lower than in the no blood system involvement group. The peripheral blood T cells CD3^+^/CD8^+^ were higher than in the no blood system involvement group (*P* < 0.05). The percentages of CD3^+^, CD3^−^/CD16^+^ CD56^+^, and CD3^−^/CD19^+^ did not differ significantly between the two groups (*P* > 0.05; Table 2).

**Table 2:** Results of peripheral blood lymphocyte subpopulations in the blood and non-blood groups of SLE (mean ± standard deviation)

Group	*n*	CD3^+^ (%)	CD3^+^/CD4^+^ (%)	CD3^+^/CD8^+^ (%)	CD4^+^/CD8^+^	CD3^−^/CD16^+^CD56^+^ (%)	CD3^−^/CD19^+^ (%)
Blood	55	70.93 ± 5.73	26.42 ± 4.43	44.51 ± 4.98	0.60 ± 0.16	7.95 ± 2.63	6.35 ± 1.83
Non-blood	25	69.88 ± 8.23	29.40 ± 5.68	39.36 ± 5.28	0.75 ± 0.12	8.00 ± 2.04	6.36 ± 2.06
*t*		0.576	−2.32	4.11	−4.693	−0.1	−0.03
*P*		0.568	0.026	0.001	0.001	0.92	0.976

### 3.4 Comparison between joint and non-joint groups

Peripheral blood T cells (CD3^+^, CD3^+^/CD4^+^) in the joint group were significantly lower than those in the non-joint group (*P* < 0.05; Table 3).

**Table 3:** Results of peripheral blood lymphocyte subpopulations in the joint and non-joint groups of SLE (mean ± standard deviation)

Group	*n*	CD3^+^ (%)	CD3^+^/CD4^+^ (%)	CD3^+^/CD8^+^ (%)	CD4^+^/CD8^+^	CD3^−^/CD16^+^CD56^+^ (%)	CD3^−^/CD19^+^ (%)
Joint	36	68.64 ± 5.27	26.69 ± 3.30	42.47 ± 5.90	0.64 ± 0.14	8.19 ± 1.86	6.17
Non-joint	44	71.5 ± 6.52	28.34 ± 5.13	42.75 ± 4.62	0.68 ± 0.17	7.91 ± 2.02	6.43
*t*		−2.17	−1.73	−0.23	−0.55	−0.49	−0.6
*P*		0.033	0.087	0.82	0.58	0.62	0.55

### 3.5 Comparison between mild, moderate, and severe activity groups of SLE

Peripheral blood T cells (CD3^+^/CD4^+^ and CD4^+^/CD8^+^) in the mild activity group were higher than in the moderate and severe activity groups. The peripheral blood T cells CD3^+^/CD8^+^ were lower than in the moderate and severe groups (*P* < 0.05). NK lymphocytes (CD3^−^/CD16^+^ CD56^+^) in the severe activity group were lower than in the mild and moderate activity groups of SLE (*P* < 0.05). Moreover, the differences in the total T lymphocytes (CD3^+^) and B lymphocytes (CD3^−^/CD19^+^) of the three groups were not significant (*P* > 0.05; Table 4).

**Table 4:** Results of peripheral blood lymphocyte subpopulations in mild, moderate, and severe active groups of SLE (mean ± standard deviation)

Group	*n*	CD3^+^ (%)	CD3^+^/CD4^+^ (%)	CD3^+^/CD8^+^ (%)	CD4^+^/CD8^+^	CD3^−^/CD16^+^CD56^+^ (%)	CD3^−^/CD19^+^ (%)
Mild active	28	70.97 ± 6.57	30.18 ± 4.54	40 ± 4.71	0.76 ± 0.13	8.75 ± 1.94	6.48 ± 2.01
Moderate active	31	70.54 ± 6.73	26.23 ± 4.67	44.16 ± 5.16	0.59 ± 0.17	8.61 ± 2.42	6.22 ± 1.99
Severe active	21	70.17 ± 6.45	25.33 ± 4.45	44.57 ± 6.54	0.58 ± 0.12	6.00 ± 2.05	6.10 ± 1.85

### 3.6 Correlation analysis of peripheral blood lymphocyte subpopulations of SLE with clinical testing indicators

**Figure 1:**
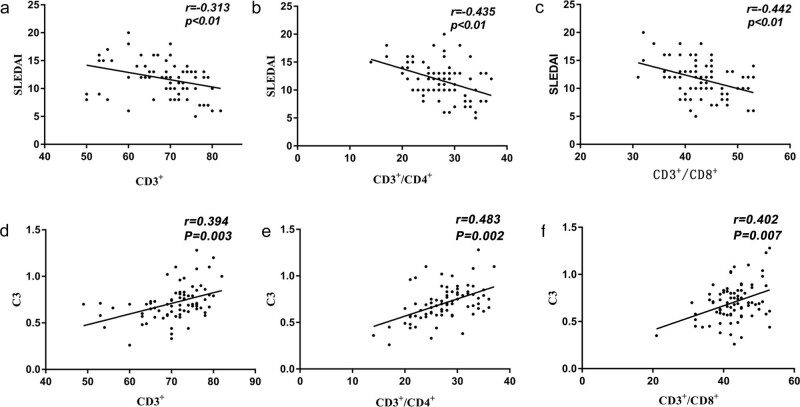
(a) Correlation analysis of peripheral blood CD3^+^ of SLE with SLEDAI; (b) Correlation analysis of peripheral blood CD3^+^/CD4^+^ of SLE with SLEDAI; (c) Correlation analysis of peripheral blood CD3^+^/CD8^+^ of SLE with SLEDAI; (d) Correlation analysis of peripheral blood CD3^+^ of SLE with C3; (e) Correlation analysis of peripheral blood CD3^+^/CD4^+^ of SLE with C3; (f) Correlation analysis of peripheral blood CD3^+^/CD8^+^ of SLE with C3.

The expression rates of total T lymphocytes (CD3^+^), helper T lymphocytes (CD3^+^/CD4^+^), and suppressor T lymphocytes (CD3^+^/CD8^+^) at the baseline level were negatively correlated with SLEDAI scores (*r*s = −0.313, *P* = 0.005; *r*s = −0.435, *P* = 0.001; *r*s = −0.442, *P* = 0.003, respectively). The expression percentages of total T lymphocytes (CD3^+^), helper T lymphocytes (CD3^+^/CD4^+^), and suppressor T lymphocytes (CD3^+^/CD8^+^) were positively correlated with C3 (*r*s = 0.394, *P* = 0.003; *r*s = 0.45, *P* = 0.002; *r*s = 0.406, *P* = 0.007, respectively) (Table 5 and Figure 1).

**Table 5:** Correlation analysis between peripheral blood lymphocyte subpopulations of SLE and clinical testing indicators

Name	CD3^+^		CD3^+^/CD4^+^		CD3^+^/CD8^+^		CD4^+^/CD8^+^		CD3^−^/CD16^+^CD56^+^		CD3^−^/CD19^+^	
	*r*	*P*	*r*	*P*	*r*	*P*	*r*	*P*	*r*	*P*	*r*	*P*
SLEDAI	−0.313	0.005	−0.435	0.001	−0.442	0.003	0.1	0.934	0.07	0.856	0.09	0.812
C3	0.394	0.003	0.45	0.002	0.406	0.007	0.163	0.215	0.149	0.186	0.177	0.283
C4	0.218	0.091	0.195	0.227	0.171	0.353	0.146	0.281	0.206	0.115	0.101	0.420
IgG	0.089	0.674	0.234	0.155	0.056	0.740	0.188	0.132	0.092	0.464	0.083	0.352
IgA	0.099	0.522	0.165	0.213	−0.147	0.257	0.203	0.071	0.167	0.322	0.134	0.264
IgM	0.141	0.381	0.066	0.582	−0.101	0.447	0.135	0.172	0.071	0.728	0.138	0.205
ESR	−0.078	0.634	−0.095	0.825	0.033	0.542	−0.055	0.796	−0.014	0.561	0.088	0.432
CRP	−0.056	0.417	−0.087	0.365	0.052	0.772	−0.044	0.625	0.095	0.518	0.069	0.481

## 4 Discussion

SLE is heterogeneous; however, the pathogenesis is unclear, and the immune system plays a critical role in SLE [1,2]. The proliferation of T and B lymphocytes inducing abnormal immune tolerance and overactivation is the main cause, and T cells play a crucial role in the overall pathogenesis of SLE [9,10]. CD4^+^ T cells mediate the activation of other immune cells in adaptive immunity [11], while CD8^+^ T cells produce toxic granules that induce apoptosis [12]. CD4^+^ T cells prompt B lymphocytes to produce immunoglobulin G antibodies that bind to self-antigens found in the glomerular basement membrane, the liver, the central nervous system, and the small blood vessels [9,10]. T cells, mainly CD4^+^ T cells, infiltrate damaged glomeruli and renal interstitium [13]. The number of infiltrating cells is correlated with renal injury, promoting apoptosis, and initiating and perpetuating the inflammatory process in LN [14]. Moreover, CD4^+^ T cells are present in the urine of patients with active LN [15]. The antigen-reactive T cells expressed by renal CD4^+^ T cells might lead to renal damage, thereby indicating the degree of inflammation of LN [16]. Gao et al. [17] hypothesized that a decrease in Treg cells decreased the number of CD4^+^ T cells. Although the proportion of CD4^+^ T cells is reduced, humoral immunity secretes a variety of cytokines to regulate cellular functions in normal immune defense [9,10]. Immune regulation is diminished, and NK cells lead to B-cell activation [1,2]. In the present study, the percentages of CD8^+^ T cells, CD4^+^/CD8^+^, and NK cells were positively correlated with C3, and it was speculated that CD8^+^ T cells and NK cells might be involved in the complement pathway.

A decrease in T cells is a risk factor for respiratory and CNS infections in SLE [18,19]. Chen and Chen [20] compared some indicators in the LN group infected with *Cryptococcal meningitis* with the uninfected group and found that the infected group had significantly decreased CD4^+^ T cells while SLEDAI was significantly higher. The low-percentage CD4^+^ T cells and high-percentage SLEDAI 2K scores are independent risk factors for SLE pneumonia [21]. Previous studies reported a significant decrease in peripheral blood CD3^+^, CD4^+^, and CD4^+^/CD8^+^ in the infected SLE group [22,23]. Abnormal peripheral blood lymphocyte immune response might lead to various infections in SLE patients [24].

The most common changes in SLE are a decrease in lymphocyte subpopulation CD4^+^ T cells and an imbalance ratio of CD4^+^/CD8^+^ [25]. Activated and senescent CD4^+^ cells in SLE blood cell subpopulations are significantly higher, and resting CD4^+^ levels are significantly lower than in healthy individuals [26]. Men et al. [27] reported a reduced ratio of CD4^+^/CD8^+^ cells in the SLE group and speculated that changes in the CD4^+^/CD8^+^ ratio interfere with and disrupt cellular immunity and lead to SLE. As confirmed previously, the absolute values of CD4^+^ or CD8^+^ T cells affect SLE progression [28,29]. Maeda et al. [30] showed that SLE CD4^+^/CD8^+^ decreased, and human leukocytes expressed the significantly associated antigen HLA-DR through CD8^+^ T cells, suggesting that the CD4^+^/CD8^+^ ratio may be an indicator to assess the treatment efficacy in some SLE patients. This study confirmed that the expression percentage of CD3^+^/CD4^+^ and CD4^+^/CD8^+^ in the LN group was lower than in the non-LN group and that of CD3^+^/CD8^+^ was higher than that in the non-LN group. The proportion of CD3^+^/CD4^+^ and CD4^+^/CD8^+^ in the blood system involvement group was lower and that of CD3^+^/CD8^+^ was higher than that in the no blood system involvement group. CD3^+^ and CD3^+^/CD4^+^ in the joint group were lower than in the non-joint group. These results are supported by Lu et al. [31], who reported that different clusters of T-lymphocyte subsets could help differentiate the different types of SLE.

Kalim et al. [32,33] proposed that the mechanism of cognitive impairment in SLE was accelerated immune senescence, and one of the markers of immune senescence was the immune risk profile (CD4^+^/CD8^+^) hypothesis. CD4^+^ T cells and CD4^+^/CD8^+^ levels were decreased, and memory function with visuospatial domains was negatively correlated with memory T cells (CD4^+^ CD45RO^+^ T cells and CD8^+^ CD28^−^ T cells), confirming that accelerated immune senescence in SLE patients led to cognitive dysfunction, especially in attention deficit, recall, and visuospatial domains. Robinson et al. [34] demonstrated that SLE CD8^+^ T cells increased with disease activity in the baseline adolescent group, with a significantly higher proportion of LN, thereby supporting the role of CD8^+^ T cells in the pathogenesis of adolescent SLE. The decreased CD4^+^ T cells, B cells, and altered immune cell subpopulations suggested that adolescent-onset SLE adapted to the dysregulated immune system. In the future, we could study the correlation between the memory function of CD4^+^ T cells, CD4^+^/CD8^+^, and visual space domain and memory T cells (CD4^+^ CD45RO^+^ T cells, CD8^+^ CD28^−^ T cells) to explore the mechanism of accelerated immune aging and cognitive impairment in patients with SLE, especially in attention disorder, memory, and visual space domain.

A close correlation was established between CD4^+^, CD8^+^, SLEDAI score, and clinical features, with the remission of SLE, ESR, SLEDAI score, and proteinuria showing a downtrend while C3 showed an uptrend and C4 showed a stable pattern. Moreover, CD4^+^ was negatively correlated with SLEDAI. CD4^+^ is a sensitive, specific, reliable, and valid indicator to reflect SLE activity and could be applied to track disease activity [28]. Zhao et al. [35] reported that CD3^+^/CD4^+^ and CD4^+^/CD8^+^ were negatively correlated with SLEDAI and positively correlated with C3 and C4, suggesting that lymphocyte subpopulations may reflect the severity of SLE activity. The comparison between SLE and healthy control groups indicated that CD3^+^/CD4^+^ and CD4^+^/CD8^+^ were negatively correlated with SLEDAI scores [23].

NK cells are rapid producers of IFN-γ and influence the adaptive immunity involved in the pathogenesis of systemic SLE [36]. Flow cytometry assays for the NK cell phenotype IFN-γ in SLE and healthy individuals resulted in a significantly lower percentage of peripheral blood NK cells in SLE patients than that in healthy individuals. A study reported that the total number of NK cells in SLE was reduced, but the number of suppressed NK cells was decreased, and the number of activated NK cells was increased, resulting in enhanced NK cell-mediated killing of normal cells [37]. The reduced number and percentage of peripheral blood NK cells in SLE, impaired cytotoxic function, differentiation, altered phenotype, changed cytokines, and NK cells involved in the pathogenesis of SLE were consistent with the results of this study. The number of NK cells was significantly increased in the SLE group and was inversely proportional to disease activity.

The present study has limitations, such as single-center, small sample size, and selection bias. Therefore, additional studies are required to substantiate the conclusion.

The immune expression abnormalities and dysregulation of T-cell subpopulations, B cells, and NK cells play a significant role in the pathogenesis of SLE. T-lymphocyte subpopulations are closely related to SLE activity, and with the popularization of the clinical application of flow cytometry, the analysis of the subpopulation could be one of the comprehensive indicators for assessing SLE activity. In addition, monitoring the changes in peripheral blood lymphocyte subpopulations is conducive to understanding the immune status of SLE patients, which is crucial for accurate diagnosis, the selection of the treatment plan, and judgment of the efficacy and safety of drug therapy.
